# Role of COX-2/PGE2 Mediated Inflammation in Oral Squamous Cell Carcinoma

**DOI:** 10.3390/cancers10100348

**Published:** 2018-09-22

**Authors:** Walaa Hamed Shaker Nasry, Juan Carlos Rodriguez-Lecompte, Chelsea K. Martin

**Affiliations:** Department of Pathology and Microbiology, Atlantic Veterinary College, University of Prince Edward Island, Charlottetown, PE C1A 4P3, Canada; wnasry@upei.ca (W.H.S.N.); jrodriguez@upei.ca (J.C.R.-L.)

**Keywords:** Oral squamous cell carcinoma, inflammation, invasion, COX-2, PGE-2, CD147

## Abstract

A significant amount of research indicates that the cyclooxygenase/prostaglandin E2 (PGE2) pathway of inflammation contributes to the development and progression of a variety of cancers, including squamous cell carcinoma of the oral cavity and oropharynx (OSCC). Although there have been promising results from studies examining the utility of anti-inflammatory drugs in the treatment of OSCC, this strategy has been met with only variable success and these drugs are also associated with toxicities that make them inappropriate for some OSCC patients. Improved inflammation-targeting therapies require continued study of the mechanisms linking inflammation and progression of OSCC. In this review, a synopsis of OSCC biology will be provided, and recent insights into inflammation related mechanisms of OSCC pathobiology will be discussed. The roles of prostaglandin E2 and cluster of differentiation factor 147 (CD147) will be presented, and evidence for their interactions in OSCC will be explored. Through continued investigation into the protumourigenic pathways of OSCC, more treatment modalities targeting inflammation-related pathways can be designed with the hope of slowing tumour progression and improving patient prognosis in patients with this aggressive form of cancer.

## 1. Introduction

Oral cancer include malignancy of the oral cavity and oropharynx, 90% of which are squamous cell carcinoma. Oral and oropharyngeal squamous cell carcinoma (OSCC) is a very aggressive neoplasm and is often diagnosed late in the disease. Extensive research has demonstrated a relationship between chronic inflammation and a variety of cancer types, including OSCC. For example, chronic hepatitis, due to hepatitis B and C viruses predispose patients to hepatocellular carcinoma [1]. Ulcerative colitis and Crohn’s disease increase the risk of colorectal cancer and gastritis associated with *Helicobacter pylori* infection has been associated with the development of gastric carcinoma [2,3]. Chronic inflammation has also been shown to play a role in the pathogenesis of oesophageal, pancreatic and gallbladder cancers [3]. Long-standing inflammation or irritation of the oral cavity caused by dental cavities and periodontitis has been also linked to the development of OSCC in some people [4]. Additionally, expression of inflammatory markers in OSCC patients has been associated with poorer prognosis [5]. Despite years of investigation focusing on inflammatory mediators in OSCC, anti-inflammatory therapy as part of OSCC treatment has been met with variable clinical success. In order to improve the prognosis of this aggressive form of cancer, it is important to increase our understanding of the mechanisms triggered by inflammation that support OSCC progression and poor patient outcomes. This review will provide a synopsis of the field’s current knowledge of OSCC biology, and will go on to summarise the mechanisms, by which inflammation contributes to disease progression and invasive behaviour, in this aggressive form of cancer.

## 2. General Epidemiology and Prognostic Factors for Oral Squamous Cell Carcinoma

OSCC is the sixth most common cancer in the world, behind lung, stomach, breast, colorectal and cervical cancer [6], with approximately 630,000 new cases and more than 350,000 deaths yearly [7]. Oral cancer is more common in developing countries compared to developed countries, with the highest percentages in Pakistan, India, Brazil, Thailand, and Slovakia [8]. In Northern America and Europe, oral cancer accounts for 5–10% of all new cancer cases with the highest incidence in France [9]. In the United States, there are approximately 41,380 new cases every year with 7890 deaths [10]. In Canada, 4700 Canadians were diagnosed with cancer of the oral cavity, and 1250 Canadians died from this disease in 2017 [11], with an overall 5-year survival rate of only 63% [12].

### 2.1. Risk Factors of OSCC

Risk factors for OSCC vary across different countries, correlating with differences in regional practices. In Western culture, the most significant risk factors are smoking and alcohol use, which appear to have synergistic roles in oral tumourigenesis. Other risk factors include alternate forms of tobacco, as well as viral infection poor oral hygiene and diet [13]. Low socioeconomic status can influence the risk of developing OSCC, but also the prognosis, since this group of patients might experience delayed medical attention, due to difficulties in accessing health care [14].

In countries, such as India and Sri Lanka, chewing betel quid (BQ, a combination of betel leaf, areca nut, and slaked lime) is a common practice, which predisposes to the development of OSCC. Addition of tobacco to BQ increases exposure to carcinogens that increase the risk of cancer [15]. Alcohol acts both independently and synergistically with tobacco in oral carcinogenesis [16]. Acetaldehyde is one of the alcohol metabolites responsible for this activity, and this compound has recently been identified as a tumour promoter [17]. These practices, through chemical and physical damage, are associated with chronic inflammation and oxidative damage to DNA (discussed in Section 3.3. Inflammation, Tumour Initiation and Promotion).

Human papillomavirus (HPV) is most well known for its causal role in anogenital cancer, but it is also associated with squamous cell carcinoma of the oral cavity, pharynx and larynx. High risk HPV, such as HPV 16 and 18, cause malignant transformation by merging its DNA into the host nuclear DNA. HPV-related OSCC is found in younger patients compared to HPV-unrelated OSCC, and is more likely to be found in patients with no history of alcohol or tobacco use [18].

Familial cancer syndromes that include Fanconi anaemia, xeroderma pigmentosa and Li-Fraumeni syndrome have been associated with development of OSCC [19]. Genetic polymorphisms have also been found to play a role in oral cancer and can be used to help determine prognosis [20]. The most common somatic (acquired) mutated genes in OSCC are p53, p16 and epidermal growth factor receptor (EGFR) [21].

### 2.2. Prognostic Indicators of OSCC

An important prognostic indicator for OSCC is tumour stage, determined using the tumour, node, metastasis (TNM) Classification of Malignant Tumours grading system, which accounts for tumour grade (which considers degree of differentiation), extension to regional lymph nodes, and presence of distant metastasis) [22]. Lymph node involvement is a particularly important predictor of the survival and prognosis [23]. With surgical treatment of OSCC, clean (tumour-free) margins of at least 5 mm are recommended in order to reduce the risk of recurrence and to improve the chances of survival [24]. OSCC readily invades into adjacent bone and eventually metastasises to the regional lymph nodes [25]. Factors that enable OSCC invasion and intravasation into lymphatics and blood vessels will be discussed in more detail later in this review, but include inflammatory mediators, such as prostaglandin E2 (PGE2) and matrix metalloproteinase (MMP) enzymes. It is important to note that exposure of broad areas of the oral cavity to the carcinogenic actions of tobacco and alcohol can lead to a new OSCC (a second primary tumour) near the original surgical site, independent of surgical margins (a phenomenon referred to as oral field cancerisation) [26].

The incidence of post-treatment dysphagia in patients with OSCC has been reported to be 40% within 3 years of treatment, and 86% of those patients will develop dangerous aspiration pneumonia [27]. The combination of dysphagia with poor nutrition, significant weight loss, and impaired immune function associated with cancer treatment often results in cachexia, fatigue, high susceptibility to infection, poor wound healing, or death [28].

## 3. Inflammation-Related Mechanisms in Cancer Pathogenesis, with Emphasis on Oral Squamous Cell Carcinoma

### 3.1. Generalities of Inflammation in Cancer

The inflammatory response is a defence mechanism initiated due to tissue injury, of any nature, through the action of a variety of inflammatory mediators. Mediators of inflammation, such as cytokines, prostaglandins, cyclooxygenase (COX) enzymes and matrix metalloproteinases (MMPs) can lead to genetic and epigenetic changes, causing suppression of tumour suppressor genes through DNA methylation and posttranslational modifications [29]. These inflammation-related mechanisms can lead to the development and progression of cancer.

Although a variety of germline mutations exist that are associated with cancer development, Aggarwal reported in 2009 that germline mutations only account for 10% of all cancers [30]. In contrast, the vast majority (90%) of cancer cases are caused by somatic mutations acquired through mechanisms related to environmental factors, such as tobacco smoking, obesity and chronic infections [30]. Studies have shown that tobacco smoke is a tumour promoter, due to its ability to initiate chronic inflammation and derivation of reactive oxygen species (ROS) [31]. 

Chronic inflammation is influenced by a wide array of factors, ranging from numerous interleukins, oxygen and nitrogen metabolites, growth factors, and lipid mediators. Many of these have been shown to not only contribute to inflammation and repair, but also to cancer progression. The focus of this review is on arachidonic acid-derived lipid mediators of inflammation, specifically PGE2. Arachidonic acid is converted into several types of bioactive eicosanoids with wide-ranging effects, through two main pathways: The cyclooxygenase pathway (generating PGE2 and other prostaglandins) and the lipoxygenase pathway (yielding a variety of leukotrienes as well as anti-inflammatory lipoxins) [32]. Products from both pathways can participate in the regulation of inflammation, but PGE2 is a dominant presence in the literature related to inflammation and cancer, including OSCC.

The importance of inflammation and PGE2 production in cancer is supported by several studies, but most notably in research showing that aspirin therapy can reduce the development of colonic cancer [33] and breast cancer [34]. Studies have shown that PGE2 and COX-2 are overexpressed in a variety of cancer types, including breast [34], OSCC [30,35], and colon cancer [33]. Regardless of the underlying cause, inflammatory responses play important roles at different stages of tumour development, including initiation, promotion, growth, invasion and metastasis [30].

**Figure 1:**
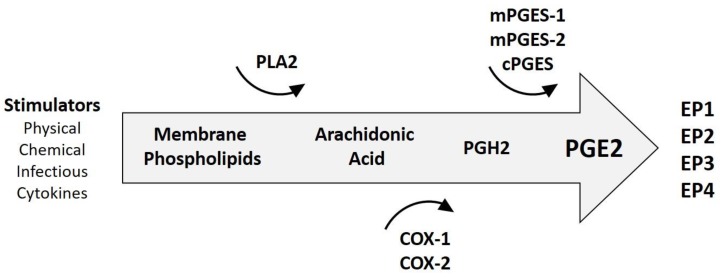
Generation of prostaglandin E2(PGE2). Stimuli, such as chemical damage, physical damage, infections and other inflammatory mediators can trigger synthesis of PGE2. The process starts with the activation of phospholipase A2 (PLA2), an enzyme, which liberates free fatty acids, such as arachidonic acid. Arachidonic acid is converted into prostaglandin H2 (PGH2) by cyclooxygenase 1 and 2 enzymes (COX-1 and COX-2). COX-1 is often referred to as the housekeeping isoform, expressed in many tissues in order to maintain homeostasis, and COX-2 is considered the inducible form, important for augmented prostaglandin synthesis when an inflammatory response is required. PGH2 is converted into prostaglandin E2 by microsomal and cytosolic PGE2 synthase enzymes (mPGES and cPGES). PGE2 elicits cellular responses through interactions with prostaglandin receptors on target cells (EP1-4).

Prostaglandin E2 (PGE2) is a mediator of many biological functions as well as active inflammation, where it promotes local vasodilatation with recruitment and activation of inflammatory cells [36]. PGE2 can be produced by all cell types, especially inflammatory cells, and triggers a range of cellular responses through binding with one or more of its four receptors (EP1, EP2, EP3 and EP4) [36,37]. Figure 1 summarises the steps in PGE2 synthesis. Outcomes of PGE2/EP receptor interactions include physiologic processes related to immune responses, blood pressure, gastrointestinal integrity and fertility. However, dysregulated PGE2 synthesis is associated with pathological conditions, such as chronic inflammation and cancer [38]. PGE2 also contributes to the healing response in chronic inflammation by promoting angiogenesis, but this also presents the undesirable effect of supporting the development of cancer, as has been demonstrated in OSCC [39].

### 3.2. Significance of Inflammation in Oral Squamous Cell Carcinoma

The role of inflammation in OSCC has been studied by many investigators. Erovic et al. (2003) found that COX-2 is expressed in OSCC tumours and the surrounding lymphocytic infiltrates, suggesting that COX-2 is an important link between chronic inflammation and carcinogenesis [40]. Further support of COX-2 in OSCC comes from Pontes et al. (2013) who found increased levels of COX-2 in oral dysplastic lesions and in OSCC, when compared with oral hyperplastic epithelium, suggesting that COX-2 is involved in the early stages of oral carcinogenesis [41]. Another study found that COX-2 is rarely expressed in normal epithelium, but it is highly expressed in dysplastic cells and carcinoma cells, and only to a variable degree in a few inflammatory cells, fibroblasts and vascular endothelial cells [42]. Chang et al. found that areca nut (a component of betel quid) is associated with increased COX-2 and PGE2 expression in human OSCC cell lines. The authors concluded that these mediators of inflammation may have a role in sustained inflammatory tissue damage and promotion of pathologic change [43]. Other studies found that COX-2 was overexpressed in dysplasia and OSCC compared with normal mucosa [44], and a COX-2 selective inhibitor reduced PGE2 production from OSCC cell lines [45].

In OSCC, the overexpression of COX-2 promotes the release of PGE2, which acts on its cell surface receptors prostaglandin E2 receptor1 (EP1), prostaglandin E2 receptor 2 (EP2), prostaglandin E2 receptor3 (EP3), and prostaglandin E2 receptor4 (EP4) to encourage the development of OSCC. [46]. Another research group found that COX-2 and PGE2 receptors are expressed in OSCC biopsy tissue and cell lines, and an EP3 receptor antagonist had an anti-proliferative effect in vitro, which was accompanied by reduced PGE2 production and cell cycle arrest [47]. Despite evidence suggesting that EP3 signalling helps support cancer progression, others have found that EP3 may actually have a protective role in certain cancers [48,49].

COX-2 and PGE2 increase migration and upregulate intercellular adhesion molecule-1(ICAM-1) expression in OSCC cells and expression of COX-2 is associated with OSCC metastases [50]. Although COX-2 expression was associated with increased levels of several types of prostaglandins, it was found that PGE2 was most important in COX-2-mediated cell migration in OSCC [50]. Further support of the role of inflammation was provided by a study that found that COX-2 and PGE2 was significantly expressed in OSCC cell lines and expression was reduced by treatment with the COX-2 inhibitor, celecoxib [51].

Interestingly, COX-2 expression represents an important marker of prognosis. Byatnal et al. found that in 58 of 75 OSCC patients, high expression of COX-2 was associated with local recurrence of the tumour, lymph node metastasis and decrease patient survival times [5]. Similarly, Pannone et al. found that higher levels of COX-2 expression are associated with poor disease-free survival [52]. Furthermore, Morita et al. found that COX-2 promotes tumour lymph-angiogenesis and lymph node metastasis of OSCC. This article is mentioned in: [53]. That research group went on to demonstrate that COX-2 causes upregulation of vascular endothelial growth factor C (VEGF-C) expression and encourages growth of new lymph vessels and subsequent lymph node metastasis [54]. Additional studies have shown that COX-2 is highly expressed in lymph node metastases and is expressed in newly formed vessels within and around the tumour, especially in the areas of tumour invasion [55].

Additional support for the role of inflammation in OSCC pathogenesis comes from studies showing a benefit when anti-inflammatory medications have been combined with cancer therapy. Combined treatment of celecoxib with cetuximab (an anti-EGFR monoclonal antibody) reduced proliferation, migration and invasion of OSCC compared to either therapy alone [56]. Similarly, Zhao et al. showed that low dose celecoxib can enhance anticancer efficacy of salvianolic acid B (the main bioactive component of *S. miltiorrhiza* in Chinese herbal medicine) through multiple mechanisms, such as induction of OSCC apoptosis and reduced proliferation along with inhibition of the COX-2/PGE2/EGFR pathway [57]. An additional benefit of combination therapy is that the dose of celecoxib can be reduced, thus lowering the risk of cardiotoxicity [57].

### 3.3. Inflammation, Tumour Initiation and Promotion

In the stepwise theory of cancer development, malignant tumours arise through a process starting with initiation, followed by promotion and continuing through a process of cancer progression. Inflammation can lead to DNA damage and mutagenesis through the action of cyclooxygenase and lipoxygenase enzymes in the AA pathway, which leads to production of lipid hydroperoxides and highly mutagenic DNA adducts [58]. Support for the role of inflammation and oxidative damage in cancer comes from patients with OSCC associated with chronic tobacco and alcoholic use. Oxidative stress is increased and antioxidant defences are decreased in patients with oral cancer [59]. There is DNA damage produced by free radicals generated by the use of tobacco [60], and this damage plays a significant role in oral carcinogenesis [60]. Nitrosative stress has also been implicated in DNA damage. Reactive nitrogen intermediates (RNI) and nitrous acid (HNO_2_) are mutagenic agents, with the potential to produce nitration, nitrosation and deamination reactions on DNA bases [61]. Nitric oxide (NO) products and NO synthase (NOS) enzymes have been found to be raised in oral cancer patients [61].

Tumour promotion is the process of tumour growth from a single initiated cell into a fully developed primary tumour [62]. Inflammation stimulates the initiated tumour cell to divide, inhibits cell death and stimulates angiogenesis, allowing the tumour to receive the blood supply necessary for growth. Inflammatory cells stimulate the production of cytokines that can stimulate expansion of initiated stem cells, causing enlargement of the cell pool that was targeted by environmental mutagens and oxidative damage and capable reconstituting the tumour after attempts to eliminate it [62]. Different studies haves shown that COX-2/PGE2 signalling can promote cancer stemness, such as in urinary bladder cancer [63], colon cancer [64], breast cancer [65] and leukaemia [66]. ROS also contribute to tumour promotion by stimulating expansion of mutated cell clones by temporarily modulating the genes related to proliferation or cell death [62], and regulate the activity of certain transcription factors, such as nuclear factor kappa-light-chain-enhancer of activated B cells (NF-κB), hypoxia-inducible factor (HIF), and p53 that control cell growth and oncogenesis [67].

STAT3 activated by the inflammatory cytokine, interleukin-6 (IL-6), and by epidermal growth factor (EGFR) [62]. Interestingly, IL-6 has been shown to be increased by PGE2 and IL-6 can, in turn, stimulate synthesis of PGE2 [68]. Upon IL-6 binding to its receptor, STAT3 translocates to the nucleus where it regulates expression of genes responsible for cell transformation, proliferation, survival, motility, and eventually the progression of malignancy [62].

NF-κB has an important role, as it inhibits apoptosis, stimulate cell proliferation, promote migratory and invasive cell behaviours [69]. The activation of NF-κB has been observed in most human cancers, such as OSCC [70], and can stimulate IL-6 expression, thus activating STAT3 and subsequent tumour growth and metastasis [62]. NF-κB can also induce the expression of COX-2. Aside from NF-κB, COX-2 can be induced by pro-inflammatory cytokines, such as interferon gamma (IFN-γ), and tumour necrosis factor alpha (TNF-α) leading to increased PGE2 production [71]. PGE2 contributes to tumour promotion through generation of ROS, stimulating oncogenic transcriptional factors, suppressing anti-tumour immune responses [72], and stimulating angiogenesis [73].

### 3.4. Inflammation and Anticancer Immunity

The microenvironment of the tumour consists of a variety of immune cells that are important for anti-tumour surveillance, including natural killer (NK) cells, cytotoxic T cells, and dendritic cells (DCs) [62]. Tumour cells can resist immune destruction for better survival by secreting immune suppressive factors or by recruiting inflammatory cells that are actively immunosuppressive, such as regulatory T cells (Tregs) and myeloid-derived suppressor cells (MDSCs) [74]. The COX-2-PGE2 signalling axis can help modulate the immune response against tumour cells. For example, PGE2 can inhibit DC activity, reduce the maturation of DCs, their ability to present antigen and activate T cells [75,76].

Macrophages can participate in anti-tumour immunity, or can help tumour progression. Macrophages originate from circulating monocytes can alter their phenotype in response to growth factors in the tumour microenvironment, causing them to develop into either M1 or M2-macrophages [77]. M1 macrophages are stimulated by IFNγ and microbial products. They also express high levels of pro-inflammatory cytokines (TNF-α, IL-1, IL-6, IL-12 or IL-23), major histocompatibility complex (MHC) molecules and inducible nitric oxide synthase, functioning to kill pathogens and prime anti-tumour immune responses [78]. In contrast, M2 macrophages, which are induced in vitro by expression of IL-4, IL-10, IL-12, IL-13, lead to suppression of the adaptive immune response, increased expression of the anti-inflammatory cytokine IL-10, and increased angiogenesis [78]. This macrophage polarisation can be influenced by COX-2 activity during monocyte differentiation, with COX-2 being the key enzyme for M2 polarisation [79]. The therapeutic significance is that COX-2 inhibitors could cause tumour associated macrophages to lose their M2 macrophage characteristics leading to reduced metastasis [79].

NK cells are a subset of lymphocytes that participate in innate immunity that secrete IFNγ and exert cytotoxic effects, including the direct killing of tumour cells. [80] PGE2 inhibits the cytotoxic functions of NK cells [81,82] preventing them from making IFNγ [83], and encourages malignant growth through successful evasion of type I interferon and T-cell-mediated death [84]. For example, in vivo inhibition of PGE2 leads to decreased lung metastasis as well as increased ability to produce IL-12 by peritoneal macrophages (a feature of the M1 phenotype) and IFNγ by spleen lymphocytes [85].

Infiltrating B cells are the main component of inflammation in some cancers, such as ductal carcinoma in situ of the breast and 20% of invasive breast tumours [86]. PGE2 has been shown to prevent the growth and division of human B cells [87].

Regulatory T cells (Tregs) are a population of T cells that maintain peripheral immune tolerance and inhibit effector T cell responses, such as cytokine production and proliferation. [88] PGE2 mediates the repressive activity of Tregs [88], and has been shown in OSCC to help the maturation of Treg cells resulting in suppressed anti-tumour immunity [89]. Another cell type that helps tumours avoid immune-mediated destruction are myeloid-derived suppressor cells (MDSCs). They are a mixed population of cells composed of myeloid progenitor cells and immature myeloid cells (IMC) [90]. These cells expand during pathological conditions, such as cancer and inflammation. They inhibit both innate and adaptive anti-tumour immunity by down-regulating macrophage cytokines, suppressing NK cell cytotoxicity, blocking cytotoxic T cell activation and regulating Treg development [91]. PGE2 helps MDSCs migrate to tumour environments, allowing the malignant cells to proliferate without interference from the host’s immune system [72].

### 3.5. Inflammation and Angiogenesis

Angiogenesis is the formation of new blood vessels and is a normal occurrence during embryonic development, organ homeostasis and disease progression [92]. Regulators for angiogenesis in cancer, including OSCC, include vascular endothelial growth factors (VEGFs) and fibroblast growth factor-2 [93] COX-2 and PGE2 production have been shown to regulate angiogenesis via vascular endothelial growth factor (VEGF), or they directly modulate endothelial cell proliferation [73]. VEGF expression is increased at the site of chronic inflammation by pro-inflammatory cytokines like IL-6, TGF-β, TNF-α and ROS. In the tumour microenvironment, low oxygen levels can stimulate induction of the HIF family of transcription factors, which promote angiogenesis and cell proliferation by causing expression of VEGF in OSCC and other cancers [94]. Studies have shown tumour hypoxia plays a significant role in the treatment resistance of OSCC [95]. A study has shown that the COX-2 pathway is related to angiogenesis by modulation of VEGF production in OSCC [39]. Interestingly, PGE2 can induce HIF-1 leading to increased expression of VEGF [96].

Tumour-associated macrophages (TAMs) are monocyte-derived cells recruited to sites of inflammation and undergo differentiation into mature macrophages within the tumour microenvironment in response to hypoxia and chemokines [97]. Studies have shown that TAMs produce factors, such as PGE2, encouraging tumour cell proliferation and formation of new blood vessels [98]. In addition to PGE2, TAMs produce proteolytic enzymes, such as MMPs that break down extracellular matrix (ECM) proteins, thus helping tumour expansion, motility and invasion [99]. Studies have found that increased TAMs are associated with poor prognosis in a majority of tumours, including OSCC [100]. Similarly, TAM depletion results in interference of angiogenesis and tumour growth that support the importance of inflammatory mediators in tumour angiogenesis [62].

### 3.6. Inflammation, Invasion and Metastasis

The leading cause of death in cancer is metastases, which requires separation of cells from the primary tumour, invasion through the basement membrane, intravasation into the blood stream, and extravasation from the blood stream at a distant site where implantation and tumour cell proliferation occur [101].

#### 3.6.1. Epithelial to Mesenchymal Transition

The process of metastasis may be explained as four major steps. The first step is epithelial to mesenchymal transition (EMT); the process of converting non-motile, polarised epithelial cells to motile, non-polarised mesenchymal-like cells, with the ability to move from a primary tumour site to distant organs, where they can establish metastases and grow [102]. The major biomarker that dominates EMT is cadherin. E-cadherin is the most important intercellular adhesion molecule in epithelial cells and has a critical role in reducing invasive behaviours of cancer cells [103]. In contrast, N-cadherin is normally expressed in neuro-ectodermal and mesodermal-derived tissue and is involved in many processes, including migration and invasion [104]. An important step in EMT is known as cadherin switching; a gain of N-cadherin and a loss of E-cadherin, which allows tumour cells to acquire motile capabilities [105]. Studies of cadherin switching in OSCC have shown that it is associated with malignant behaviour, including invasiveness, poor differentiation and lymph node metastasis [106].

Inflammatory cytokines (IL-6, TGF-β) and transcription factors (Twist, Snail and STAT3) are important regulators of EMT and metastasis. Twist and Snail are transcription factors that inhibit E-cadherin expression in epithelial cells [107]. TGF-β is a cytokine that modulates inflammation and activity of stromal and immune cells. It is produced by cancer cells, myeloid cells, and T lymphocytes and has a role in EMT development [108]. 

Wendt et al. recently reviewed the role of the IL-6 in the development of EMT [109]. Studies showed IL-6 expression and its activation of STAT3 have been associated with development and progression of carcinomas. Expression of Twist or Snail can stimulate the production of IL-6, leading to activation of STAT3, which in turn is responsible for EMT [110]. Overexpression of matrix metalloproteinase 1 (MMP1) by cancer cells in OSCC is essential for the process of EMT [111]. Once activated, the majority of MMPs function to break down collagen (types I, II, and III), as well as releasing growth factors and peptides from the extracellular matrix [112]. MMP expression in patients with advanced stage of cancer was linked to decreased survival [113]. The relationship between inflammation and tumour invasion will be discussed in more detail below.

#### 3.6.2. Tumour Invasion

Following the development of invasive behaviour, cancer cells undergo the second step of metastasis where they intravasate into blood vessels and lymphatics, which is promoted by inflammation through the production of mediators that increase vascular permeability [114]. Intravasation is regulated by MMPs [115], and PGE2 [116]. PGE2 can stimulate production of lymphangiogenic growth factors (VEGF-C and VEGF-D), which play important roles in regulating of the patency of collecting lymphatic vessels draining the tumour and facilitating metastasis [117].

One of the ways that tumour cells survive is by tumour cell-induced platelet aggregation (TCIPA) [118]. platelets are activated by the interaction with cancer cells causing release of mediators, such as COX-2 [119]. Dovizio et al. showed that expression of COX-2 can be induced by interaction of platelets with colon carcinoma cells [120]. Platelet aggregation protects tumour cells from immunological attack in the circulation by protecting them from natural killer (NK) cells [121].

The third step of metastasis is extravasation, in which the tumour cells leave the vasculature at the metastatic site. Several studies have shown that the preferences of circulating tumour cells (CTCs) to specific organs is regulated by genetic alterations in the cancer cells [122], and by the types of mediators produced by the tumour cells. Many malignant cells upregulate expression of chemokine receptors during pre-malignancy, so they can recognise new anatomic sites by local chemokine production and specific molecular signals displayed on the vascular endothelium [123].

Tumour cells have to squeeze between endothelial cells to extravasate from the blood vessels to enter the new tissues. First, they attach to the luminal side of endothelial cells (ECs), which is mediated by different adhesion receptors on the tumour and ECs and is also facilitated by platelets. Upon activation, platelets produce mediators that affect both vascular permeability and tumour cell adhesion to the endothelium; these include VEGF, and MMPs [124].

In the fourth step, newly arrived cancer cells settle into their metastatic site where they interact with immune, inflammatory and stromal cells and start to proliferate [125]. The processes of local invasion, angiogenesis and repeated rounds of metastasis can continue through the mechanisms described above, contributing to ever increasing tumour burden and deteriorating health of the cancer patient.

#### 3.6.3. The Role of CD147 in Inflammation and Invasion

The connection between tumour cells, non-malignant stromal cells and extracellular matrix (ECM) of the tumour microenvironment helps the tumour to invade locally and later metastasise to different sites [126]. ECM consists of collagen, fibronectin, elastin, and proteoglycans. The ECM is subjected to reorganisation and breakdown during processes, such as wound healing, inflammation, and organogenesis [127].

Different groups of proteolytic enzymes are involved in matrix breakdown, but the matrix metalloproteinase (MMP) group of enzymes is the most important in tumour invasion and metastasis [128]. MMPs are zinc-dependent endopeptidases, which facilitate tumour invasion and modulate tumour associated angiogenesis. They are classified according to a combination of amino acid sequence, peptide domain structure, and substrate specificity into four main groups of MMPs, which are the collagenases, gelatinases, stromelysins and membrane-type MMPs [129,130]. There are twenty-five different MMPs in mammals, of which twenty-three are found in humans [131]. MMPs have been shown to concentrate on membranous projections of tumour cells (invadopodia) that allow the cell to invade into adjacent stroma and ultimately gain entrance to the vasculature and finally establish metastatic disease [132,133,134].

Studies have shown that MMP2 expressed by stromal fibroblasts help the growth of pulmonary metastases in breast cancer [135]. While in colorectal cancer, MMP13 has an important role in promoting the growth of liver metastasis [136]. In OSCC patients, MMP-7 expression is associated with metastases and poor outcome [137], while MMP-9 is associated with reduced survival [138]. Research has shown that curcumin treatment inhibits invasiveness in oral cancer by reducing the expression of MMP-2 and MMP-9 [139]. Furthermore, the invasion of OSCC into adjacent bone might be due to increased expression of MMP-1, MMP-9, and CD147 (an MMP activator) [140]. MMP and CD147 are associated with bone invasion [141]. Studies have shown that in colorectal cancer, MMP-11 and CD147 increase the progression of the disease [142], and act as prognostic factors as they affect the survival rate [143]. In tumour cells, CD147 stimulates the formation of MMPs and participates in invasion [140].

CD147, also referred to as Basigin and extracellular matrix metalloproteinase inducer (EMMPRIN), is a glycosylated protein belonging to the immunoglobulin superfamily. [144] CD147 presents in two forms; the transmembrane and soluble forms. The transmembrane part consists of 2 segments, an extracellular domain and a cytoplasmic tail [144], that plays an important role in induction or stimulation of MMP, while the soluble part has been shown to be a useful marker for hepatocellular carcinoma [145]. CD147 analogues have been identified in different species, such as Basigin and M6 leukocyte activation antigen in people [146], MRC-OX47 antigen/CE9 in rat [147], and blood–brain barrier specific HT7 molecule in chickens [148].

An association between increased CD147 expression and poor prognosis has been demonstrated in a variety of cancers, such as OSCC [149], colon cancer [142], and breast cancer [150]. CD147 activation of MMPs in the tumour microenvironment helps tumour cells undergoing EMT to invade the surrounding stroma [151]. Studies found that combination therapy of an antiangiogenic drug and anti-CD147 may be helpful in targeting angiogenesis, because CD147 acts as a co-receptor for VEGF in vitro and in vivo [152]. It has also been demonstrated that CD147 has a role in cancer cell metabolism and proliferation through effects on glucose metabolism and inhibition of the p53 pathway [153].

CD147 is expressed at a low level on resting monocytes, neutrophils and lymphocytes, but its expression is upregulated in activated neutrophils, macrophages, monocytes, T lymphocytes and dendritic cells, illustrating its role in inflammation and immune response [154,155]. Additional studies have shown that CD147 has a role in immune development as, its expression on thymocytes is increased compared to mature peripheral blood T cells [156]. The extracellular domain of CD147 acts as a receptor for cyclophilins, serving as a chemoattractant for neutrophils and T lymphocytes [157]. Binding of cyclophilins to CD147 results in leukocyte recruitment, allowing CD147 to contribute to inflammatory disease [157], such as brain injury after trauma, multiple sclerosis [158], acute lung injury [159], allergic asthma [157], and rheumatoid arthritis [160]. It is used as a marker in combination with others in lupus nephritis as it is elevated in renal damage [161]. CD147 is known to play a role in the pathogenesis of Alzheimer’s disease [162], myocardial infarction [163], and human immunodeficiency virus-1 (HIV-1) [164].

A study showed fluvastatin (a statin) can inhibit CD147 expression from macrophages in coronary atherosclerosis, further supporting the relationship between inflammation and CD147 [165]. CD147 is present at low levels on resting platelets, while it is upregulated on activated platelets [166]. It helps platelet-monocyte interaction leading to release of inflammatory mediators from monocytes, such as IL-2 and MMP9 via activation of NF-κB pathway inside the monocytes [167]. Interestingly, a study has shown a relationship between CD147, COX-2 and inflammation, where COX-2 can modulate the production of CD147 via a PGE2-dependent pathway in macrophages and that the effect can be inhibited by a COX-2 inhibitor in atherosclerotic plaques [168]. Angiotensin II can up-regulate CD147 expression in macrophages, via type 1 angiotensin receptor and the COX-2/PGE2 signal transduction pathway, and the effect can be inhibited by losartan (an angiotensin II antagonist) [169]. With regard to cancer, a study using a mouse model of OSCC revealed that CD147 inhibition resulted in decreased inflammatory mediators, such as IL-6 along with reduced collagen degradation and cell growth in vitro [170]. In hypopharyngeal carcinoma, both COX-2 and CD147 were expressed and associated with tumour invasion, lymph node metastasis and survival, suggesting that COX-2 and CD147 are important for prognosis [171]. There has been great interest in developing CD147 monoclonal antibody therapy for cardiovascular and other inflammatory conditions, raising the possibility that anti-CD147 therapy may also be helpful for the treatment of inflammation-related invasive cancer. Anti-CD147 therapy has been attempted experimentally in mice bearing OSCC xenografts, revealing that mice treated with anti-CD147 had significant tumour growth delay when compared with untreated controls [170]. Another strategy for targeting invasion is through the use of MMP inhibitors, but early programs targeting MMPs failed in clinical trials, posing a challenge to their approval as a form of cancer treatment. [172,173] Despite evidence suggesting an interaction between COX-2-mediated inflammation and CD147, the presence of such interaction has not been studied in OSCC and warrants further attention.

## 4. Anti-Inflammatory Drugs in Cancer Prevention and Treatment

Non-steroidal anti-inflammatory drugs (NSAIDs) have been shown to suppress cell proliferation and carcinogenesis through different mechanisms. COX-dependent mechanisms of NSAID-suppression of breast cancer were demonstrated in human cell lines and in mice, when the inhibitory effect of NSAIDs was eliminated after genetic knockdown of COX-2 [174].

There is an important link between NSAIDs and reduced cancer mortality as well as reduced rates of primary and recurrent cancer. Studies have proposed that the use of aspirin and NSAIDs decrease the incidence of breast cancer [34]. Colorectal cancer and lung cancer patients using NSAIDs over long periods had decreased mortality rates compared to non-NSAID users [33,175]. Using sulindac (a type of NSAID) has been shown to decrease the number and recurrence of colonic polyps in familial adenomatous polyposis (FAP) patients [175]. A specific COX-2 inhibitor (celecoxib) may have activity in the treatment of high-grade cervical dysplasia [175]. Anti-inflammatory drugs have also been used as adjuvant therapy in the treatment of prostate and lung cancer [175], and have been shown to increase apoptosis and suppress the proliferation in nasopharyngeal carcinoma [176].

Administration of anti-inflammatory agents can decrease the toxicity of chemotherapeutic agents [177]. For example, in patients with refractory metastatic prostate cancer, administration of celecoxib with docetaxel (anti-mitotic chemotherapy) decreased hematologic toxicity [178]. Addition of celecoxib to a FOLFIRI (folinic acid, fluorouracil and irinotecan) regimen therapy decreases chemotherapy-related diarrhoea [179]. Additionally, administration of anti-thrombotic NSAIDs with chemotherapy helps prevent thromboses in cancer patients, allowing cytotoxic agents to access small tumours and improve the prognosis of patients with ovarian cancer [180]. Celecoxib combined with erlotinib (a receptor tyrosine kinase inhibitor targeting EGFR) and radiation therapy are active combinations in patients with recurrent OSCC [181]. However, various adverse effects have been associated with the use of NSAIDs. These include gastrointestinal ulceration [182] and impaired platelet function linked with COX-1 inhibitors, as well as cardiovascular and kidney toxicity associated with prolonged use of selective COX-2 inhibitors [183]. It has been suggested that adding a protein pump inhibitor to traditional NSAIDS can reduce adverse effects in the gastrointestinal tract, and patients at risk for cardiovascular events would benefit from replacing COX-2 specific inhibitors with non-specific NSAIDs in order to spare the cardiovascular system [184]. The US Food and Drug Administration approved Yosprala (a combination of aspirin and omeprazole, a type of proton pump inhibitor) to decrease incidence of GI toxicity in patients requiring long term NSAID therapy for prevention of heart attack and stroke [185].

## 5. Conclusions

**Figure 2:**
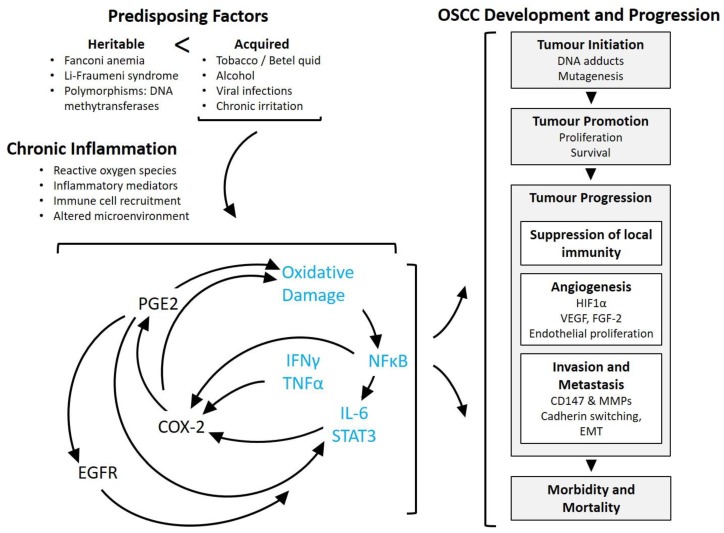
General role of PGE2-mediated inflammation in oral and oropharyngeal squamous cell carcinoma (OSCC) development and progression. Most cases of OSCC are attributable to acquired risk factors that are dominated by long-term tobacco and alcohol use, and infection with high risk human papillomavirus (HPV), rather than germline mutations. Infection and mechanical or chemical factors leads to chronic inflammation accompanied by oxidative damage and genetic and epigenetic alterations. In general, chronic inflammation is driven by a cycle of increased expression of mediators, including (but not limited to) PGE2, IFNγ and TNFα; arising from immune cells, stromal cells and tumour cells. PGE2 synthesis is chiefly attributed to COX-2 in OSCC. COX-2 activity and PGE2 have been shown to contribute to oxidative damage, increased expression of inflammation-associated transcription factors, and altered gene expression. Demonstrated outcomes of COX-2/PGE2 activity include activation of EGFR signalling, increased HIF1α and VEGF, and increased activity of CD147 and MMP enzymes. Collectively, chronic inflammation contributes to OSCC at various stages from initiation of carcinogenesis through invasion and metastasis, ultimately contributing to patient morbidity and mortality. Blue text signifies hypothesised mechanisms based on studies in other forms of cancer. COX-2 (cycloxygenase-2), PGE2 (prostaglandin E2), EP1-4 (PGE2 receptors 1 though 4), NFκB (nuclear Factor kappa-light-chain-enhancer of activated B cells), IL-6 (interleukin 6), STAT3 (signal transducer and activator of transcription 3), IFNγ (interferon γ), TNFα (tumour necrosis factor α), EGFR (epidermal growth factor receptor), HIF1α (hypoxia inducible factor 1α), VEGF (vascular endothelial growth factor), FGF-2 (fibroblast growth factor 2), CD147 (cluster of differentiation factor 147), MMP (matrix metalloproteinase), EMT (epithelial to mesenchymal transformation).

The concept that infectious agents and inflammation contribute to cancer risk has been widely accepted, translating into preventative recommendations, such as condom use to prevent HPV transmission, screening blood products for hepatitis B, C and HIV infections, developing vaccination programs against hepatitis B and HPV infection, and educating the public about smoking cessation. In order to reduce the incidence of OSCC, health professionals must educate those at risk about the predisposing factors and habits associated with cancer, the early signs of the disease, and the complications of delaying seeking medical advice [186]. This is important because reducing tobacco, betel quid and alcohol consumption, along with increasing antioxidant consumption through dietary fresh fruits and vegetables can reduce the incidence of oral cancer [186,187]. To increase the rate of early diagnosis of OSCC, healthcare practitioners should examine the mouth as part of a general examination. Chronic inflammation is linked to OSCC by different inflammatory mediators that support tumour initiation, progression, invasion and finally metastases (Figure 2). Continued research focusing on blocking inflammation-related mechanisms in cancer, such as the COX-2 / PGE2 pathway and CD147, may help to decrease tumour formation and progression and thus potentially improve the quality of life and survival rates of cancer patients, including those with OSCC.
